# Convulsion and Atrial Fibrillation after Transforaminal Cervical Epidural Lidocaine Injection

**DOI:** 10.5811/cpcem.2018.9.39539

**Published:** 2018-10-16

**Authors:** Ashley E. Carter Powell, Aimee Mishler, Dan Quan

**Affiliations:** * Maricopa Integrated Health System, Department of Emergency Medicine, Phoenix, Arizona; † University of Arizona College of Medicine - Phoenix, Phoenix, Arizona; ‡ Banner - University Medical Center Phoenix, Department of Medical Toxicology, Phoenix, Arizona; § Creighton University, School of Medicine, Phoenix, Arizona

## Abstract

Lidocaine has been widely used as a local anesthetic as well as an antiarrhythmic. Its use in epidural anesthesia is increasing, which has introduced new risk and potential for harm not associated with older indications. We present a case of convulsion and atrial fibrillation seen after transforaminal cervical epidural injection with two milliliters of 2% lidocaine (40 milligrams) that resolved with no long-term sequelae. Patient had a negative serum lidocaine level. With cervical epidural injections being a common treatment for radicular pain, it is important for medical providers to be aware of the various complications associated with this procedure.

## INTRODUCTION

Lidocaine quickly grew in popularity among local anesthetics when it came to market in 1948 due to its quick onset of action and safer side-effect profile.^1^ Over time, new uses for the drug have come to light. Lidocaine is well known for treatment of ventricular dysrhythmias and has become a popular agent for use in epidural anesthesia. New indications and uses introduce new risk and potential for harm. As a Class 1B antiarrhythmic, lidocaine blocks voltage-gated sodium channels, which inhibits both initiation and conduction of nerve impulses by decreasing neuronal membrane permeability to sodium ions.^1^ Adverse effects typically result with systemic toxicity in a dose-related response and primarily affect the central nervous system (CNS) and the heart, ranging from mild neurologic symptoms to seizures and, in severe cases, cardiac arrest.^2^–^4^

Serum lidocaine levels can be measured to monitor for toxicity in patients undergoing continuous infusion or in cases where toxicity is suspected. However, these laboratory tests are not readily available and are not widely used or particularly clinically helpful. Over the past several decades, cervical epidural injections with both local anesthetics and corticosteroids have become a mainstay of treatment for radicular neck and back pain.^5^,^6^ Complications of these procedures are not common but can be devastating when they occur.^5^–^7^ We present a case of CNS and cardiotoxicity effects seen during cervical epidural injection. With cervical epidural injections being a common treatment modality for radicular neck pain, it is important for medical providers to be aware of the potential complications associated with this procedure and their presentations.

## CASE REPORT

A 51-year-old male weighing 131 kilograms (kg) presented to the emergency department (ED) via ambulance with altered mental status and slurred speech after undergoing cervical epidural injection with two milliliters (mL) of 2% lidocaine (40 milligrams [mg]) under fluoroscopic guidance in an ambulatory setting. He became unresponsive during the injection with subsequent brief convulsive activity for which he was given 2 mg of midazolam. Emergency medical services was called and found him obtunded with shallow breathing and low oxygen saturations requiring ventilation assistance. On arrival to the ED his breathing was spontaneous and erratic with low oxygen saturations. He remained somnolent with slurred speech, unable to answer questions appropriately or follow commands. Preparations were made for intubation given altered mental status and low oxygen saturations; however, oxygen saturations and mental status improved within the first 10 minutes of arrival and ultimately intubation was not required.

On cardiopulmonary monitor he was noted to have an irregularly irregular heart rhythm. Electrocardiogram showed atrial fibrillation with a rate of 82 beats per minute. Hemodynamically he was stable. He converted to normal sinus rhythm 20 minutes later. He reported no history of atrial fibrillation. Within 60 minutes of ED arrival the patient’s mental status was back to baseline without recollection of the events that had occurred after the start of the procedure. He only had chest wall pain, possibly from sternal rub or from any bystander chest compressions that may have been performed when he became unresponsive. Imaging studies obtained included the following: chest radiograph, computed tomography (CT) of the head, CT angiogram of the head and neck, and CT of the chest with intravenous contrast. No pertinent imaging abnormalities were identified. Serum/plasma levels of lidocaine and its primary active metabolite, monoethylglycinexylidide (MEGX) were obtained 15 minutes after patient arrival. Both levels returned undetectable. The patient was observed overnight in the hospital and remained asymptomatic and without any further dysrhythmia. He was discharged home the following day on aspirin 325 mg daily and with a referral to outpatient cardiology.

## DISCUSSION

Symptoms of lidocaine toxicity typically occur after excessive dosage or inadvertent intravascular injection, with the latter being more common in the case of local anesthetic toxicity.^4^ Adverse effects of lidocaine toxicity include perioral numbness, visual and auditory disturbances, drowsiness, slurred speech, twitching, loss of consciousness, tonic-clonic seizures, respiratory depression and cardiac arrest.^2^–^4^ The severity of CNS symptoms generally correlate with serum lidocaine level, with the risk of seizure and respiratory depression increasing as dosage and serum lidocaine level increase.^4^,^8^ A serum lidocaine level of greater than five micrograms (mcg)/mL is considered to be representative of toxicity resulting in CNS manifestations.^3^ Despite this relationship, toxicity has also been demonstrated in animal models at smaller dosages, attributed to retrograde passage of local anesthetic under pressure (reverse intracarotid flow), transarterial diffusion during stellate ganglion block, and inadvertent injection into arterial vasculature during dental procedures.^4^,^9^,^10^ This has not been demonstrated in human subjects; however, we propose a similar mechanism as an explanation in this case by which toxicity occurred at a smaller-than-expected dosage in our patient via inadvertent intravascular injection in close proximity to the CNS.

Our patient exhibited typical CNS symptoms of lidocaine toxicity during and directly after attempted cervical epidural injection with 2 mL of 2% lidocaine including tonic-clonic seizure activity, respiratory depression, drowsiness and slurred speech. His lidocaine and MEGX levels returned undetectable, as we would expect after administration of 40 mg of lidocaine in a 131-kg male. There are no reports in the literature regarding the minimum dosage of lidocaine required to obtain a detectable serum level. However, the patient’s serum lidocaine concentration can be estimated using the dosage he received (40 mg) and the reported volume of distribution (Vd) for lidocaine in the literature. The Vd for lidocaine varies between individuals but is reported between 0.6–4.5 L/kg.^11^ Using the equation, dose divided by Vd equals serum concentration (D/V = C), we were able to estimate our patient’s serum lidocaine concentration at the time of injection. Assuming complete and immediate absorption, given we suspected intravascular injection, and using the most conservative Vd available (0.6 L/kg), our patient’s serum lidocaine concentration would have been no more than 0.51 mcg/mL at the time of injection. The reporting limit for detection of both lidocaine and MEGX on the tests performed was 0.5 mcg/mL. Thus, it is possible that even if the levels had been obtained immediately after injection they may have returned undetectable.

**CPC-EM Capsule**

What do we already know about this clinical entity?

Lidocaine toxicity can occur with excessive dosage or inadvertent intravascular injection. Common signs and symptoms involve the central nervous system and cardiovascular system.

What makes this presentation of disease reportable?

Our patient received a small dose of lidocaine, not expected to cause toxicity, and exhibited classic symptoms of toxicity and atrial fibrillation, which has not previously been described.

What is the major learning point?

Even small doses of lidocaine, which are considered therapeutic, may cause toxicity if inadvertently injected into an intravascular space.

How might this improve emergency medicine practice?

Lidocaine toxicity is a rare complication of a common procedure, epidural injection. It is important to be aware of this complication and the presentation of lidocaine toxicity.

The elimination half-life of both lidocaine and MEGX is approximately 30–90 minutes.^12^ Our patient’s lidocaine and MEGX levels were not obtained until approximately 30–40 minutes after the time of injection, further decreasing the likelihood of obtaining detectable levels. Typically, this dosage of lidocaine would not be expected to cause toxicity. We believe his CNS levels at the time of injection were likely elevated above the threshold to cause symptoms of toxicity secondary to inadvertent intravascular injection, and his symptoms support this hypothesis. Sharma et al. described a similar case of CNS symptoms after inadvertent intravascular injection with only 1 mL of 2% lidocaine (20 mg) into an aberrant carotid artery overlying the trachea during a percutaneous dilation tracheostomy. No lidocaine or MEGX levels were obtained; however, inadvertent intravascular injection was confirmed with aspiration of blood after symptom onset during injection. Arterial pulsation on magnetic resonance angiography revealed aberrant carotid artery overlying the area of injection.^13^

In addition, our patient exhibited atrial fibrillation, which to our knowledge is a dysrhythmia that has not been previously described with lidocaine cardiotoxicity. Direct cardiotoxicity has been shown to require higher serum lidocaine levels than required to produce CNS toxicity.^4^,^8^ Lidocaine has commonly been used to treat ventricular dysrhythmias; however, in toxicity, myocardial depression, bradycardia, dysrhythmias, atrioventricular blocks as well as acceleration of ventricular response in atrial tachydysrhythmias have been reported.^3^,^4^,^8^ Lidocaine depresses automaticity; thus, atrial fibrillation seems an unlikely dysrhythmia to occur. Given the ability of lidocaine to depress the sinus node, it is possible that atrial fibrillation occurred in response to suppression of the sinus node’s automaticity in the setting of acute lidocaine toxicity. It has also been proposed that the CNS may play a role in cardiotoxicity; specifically, the exposure of the medulla to local anesthetics seems to result in cardiovascular changes.^4^ This appears to be most profound with bupivacaine and has not been previously associated with atrial fibrillation. Other factors that may contribute to cardiovascular changes in the setting of lidocaine toxicity include hypoxia, hypercarbia and acidosis^4^,^7^ and our patient may have suffered mild hypoxia for a period of time. Our patient’s episode of atrial fibrillation was self-limited and rate-controlled without pharmacologic intervention, correcting to normal sinus rhythm within 30 minutes.

There are two common approaches for delivery of cervical epidural injections for treatment of radicular pain: transforaminal and interlaminar.^7^ Our patient underwent transforaminal cervical epidural injection via anterolateral approach.^14^ All epidural injections carry risks including increased radicular pain, vasovagal reaction, intravascular injection, vascular injury, dural puncture, spinal cord injury, and spinal cord infarction.^5^,^7^,^15^ While transforaminal injections are considered superior in that they deliver medication to a more-specific anatomical area, this technique harbors increased risk of inadvertent intravascular injection and injury.^5^,^7^ Additionally, multiple studies have identified cervical epidural injections as having higher risk for inadvertent intravascular injection when compared to thoracic, lumbar, and sacral epidural injections.^7^,^10^ We surmise that our patient’s symptoms were the results of acute lidocaine toxicity from inadvertent intravascular injection while undergoing transforaminal cervical epidural injection.

## CONCLUSION

Epidural injections are commonly used to treat radicular pain, and while complications are rare they can be devastating. Lidocaine is often used as a treatment medication or as a test injection prior to corticosteroid injection. Lidocaine toxicity can range from subtle to severe CNS and cardiotoxic effects, or even death. While serum lidocaine and MEGX levels can be obtained to evaluate for toxicity they are not readily available. We present a novel case of CNS and cardiotoxicity after suspected inadvertent intravascular injection of a small dose of lidocaine during cervical epidural injection with negative serum lidocaine and MEGX levels. It is important to be aware of the various complications of epidural injections, how they present, and the limitations of laboratory studies for diagnosis of lidocaine toxicity.

Documented patient informed consent and/or Institutional Review Board approval has been obtained and filed for publication of this case report.
